# Feasibility of a Facebook Intervention for Exercise Motivation and Cardiac Rehabilitation Adherence: Study Protocol

**DOI:** 10.2196/resprot.7554

**Published:** 2017-08-18

**Authors:** Lee Anne Siegmund, Haitham M Ahmed, Michael Todd Crawford, James Frank Bena

**Affiliations:** ^1^ Nursing Institute Office of Nursing Research and Innovation The Cleveland Clinic Cleveland, OH United States; ^2^ Heart and Vascular Institute Department of Cardiovascular Medicine The Cleveland Clinic Cleveland, OH United States; ^3^ Lerner Research Institute Quantitative Health Sciences The Cleveland Clinic Cleveland, OH United States

**Keywords:** cardiac rehabilitation, social media, adherence, motivation, Facebook, Self-Determination Theory, Behavioral Regulation in Exercise Questionnaire-3, BREQ-3, Psychological Need Satisfaction in Exercise scale

## Abstract

**Background:**

While cardiac rehabilitation has been shown to be effective at improving coronary heart disease (CHD), participation is generally poor. Attempts to increase uptake and adherence often fail. Use of a Facebook intervention for this population may be a unique opportunity to support self-determined motivation and affect adherence.

**Objective:**

To evaluate the impact of a Facebook intervention on motivation for exercise and adherence to cardiac rehabilitation in patients with CHD during a 12-week, Phase II cardiac rehabilitation program.

**Methods:**

A prospective, randomized controlled pilot study, grounded in Self-Determination Theory, will be conducted. Participants will be recruited from inpatient, or the intake visit to outpatient, cardiac rehabilitation, and then randomly assigned to the intervention or comparison group. Participants in the intervention group will take part in a private Facebook group. Weekly posts will be designed to support self-determined motivation, measured at baseline and postcardiac rehabilitation by the Behavioral Regulation in Exercise Questionnaire-3 (BREQ-3). The Psychological Need Satisfaction for Exercise (PNSE) scale will measure fulfillment of needs that affect motivation. Participants in the comparison group will be given the same materials, but these will be supplied via handouts and email. The number of sessions attended will be tallied and analyzed using *t* tests. Overall motivation will be evaluated using analysis of covariance (ANCOVA) models. Multivariate analysis of variance models will be used to evaluate differences in the change across motivation subtypes. If significant, ANCOVA models for each subtype will be fit. ANCOVA models will be used to compare changes in needs satisfaction, overall and separately among the three subscales, between groups. Engagement in the Facebook group will be measured by number of “likes” and self-report of weekly visits to the group.

**Results:**

This project was funded in July 2017 and recruitment is currently underway. The recruitment goal is 60 cardiac rehabilitation patients. Data collection is anticipated to be complete by July 2018.

**Conclusions:**

This pilot study will be the first to examine the effect of a Facebook intervention on patient adherence and motivation for exercise in a cardiac rehabilitation setting. Engagement in the Facebook group and participation in the study will help to determine the feasibility of using Facebook to affect adherence and motivation in cardiac rehabilitation patients, potentially improving outcomes through the use of a unique intervention.

**Trial Registration:**

ClinicalTrials.gov NCT02971813; https://clinicaltrials.gov/ct2/show/NCT02971813 (Archived by WebCite at http://www.webcitation.org/6sRsz8Zpa)

## Introduction

### Background

Coronary heart disease (CHD) is the leading killer of men and women and currently accounts for 15.5 million cases in the United States [1]. Phase II cardiac rehabilitation, a Class 1 recommendation by the American College of Cardiology Foundation and the American Heart Association, is a secondary prevention program that has been shown to be safe and effective in treating patients diagnosed with existing CHD [2-10]. However, despite the reported effectiveness of cardiac rehabilitation, many at high risk for CHD are less likely to adhere to the program [11]. Utilization of cardiac rehabilitation is low overall, particularly for women, minorities, and those with comorbidities [4], and attempts to increase uptake and adherence often fail [12].

In recent years, Web-based interventions have been used to examine exercise adherence and theory-supported apps have enabled feedback on exercise intensity and adherence in remotely delivered cardiac rehabilitation [13]. Interventions utilizing the Web improved daily step counts [14] and physical activity intensity [15]. The use of such apps has been shown to be feasible and acceptable for use in special populations, including patients with cystic fibrosis [16] and cancer survivors [17]. A recent randomized controlled trial utilizing online social media to test its effect on physical activity found that the social support provided by the program resulted in an increase in group cohesion [18]. The perception of group cohesion may be important to patients in cardiac rehabilitation since social support was found to be an important component in exercise adherence [19]. Due to the vital role that social support has played in helping people to become more self-motivated [20], it is appropriate to examine unique ways to foster a sense of belonging or connectedness.

Social media is growing in popularity, making it an interesting venue for delivery of an intervention designed to affect cardiac rehabilitation adherence. Facebook in particular has the most engaged users of all social media sites, with more than 75% logging in daily [21]. Use of social network platforms on the Web, such as Twitter or Facebook, has helped patients manage personal health and has increased adherence to medical treatment [22], possibly through a sense of involvement and social support. Joseph et al [15] showed pilot data that supports Facebook as a tool for promoting physical activity by utilizing education and group discussions. Facebook, relative to other social media or Web-based interventions, has been reported to have high retention rates when used to affect health behaviors [23]. While Facebook has been studied as a means to improve physical activity in a number of populations [17,24-27], there is a knowledge gap regarding the effectiveness of social networking interventions used to promote health [28] and its use in cardiac rehabilitation as a tool to improve motivation.

### Theoretical Framework

**Figure 1:**
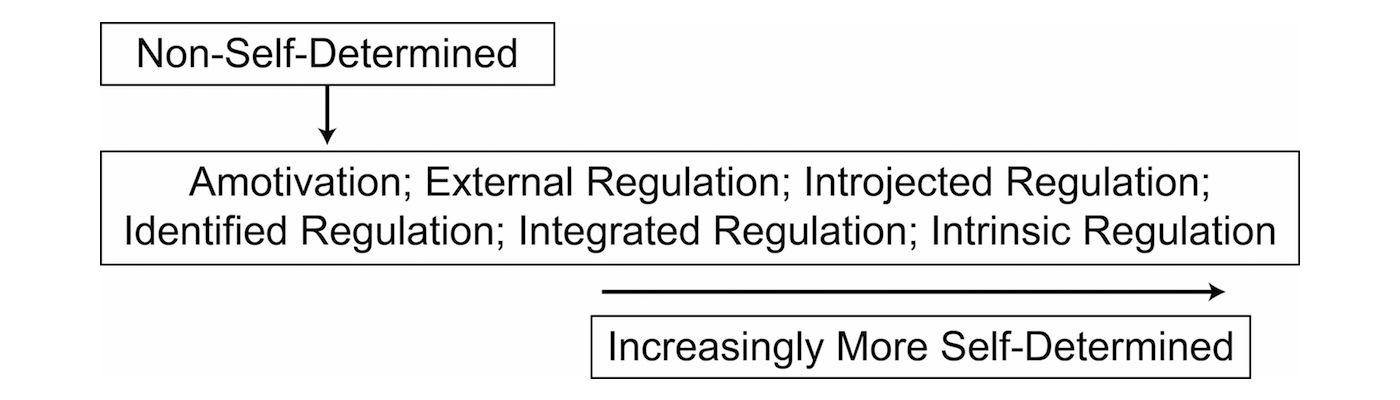
Self-Determination Theory.

This study is grounded in Self-Determination Theory [20,29,30], which defines motivation in terms of intrinsic and extrinsic sources (see Figure 1). Self-Determination Theory focuses on social and cognitive factors and how those factors influence an individual’s motivation. The theory describes motivation as being on a continuum with behavioral regulators ranging from amotivation, in which a person lacks intention to do an activity, to intrinsic regulation, in which an individual may do the activity simply for the joy of it [20]. Self-Determination Theory specifically examines conditions that lead to self-determined (ie, internalized) motivation and states that three psychological needs are necessary for it to exist: competence, autonomy, and relatedness [20]. A motivationally supportive environment supports these three needs in several ways. Competence, in essence self-efficacy, can be supported through provision of structure, offering participants positive feedback and helping them to set realistic goals [31-33]. Competence, according to Cognitive Evaluation Theory, a subtheory of Self-Determination Theory, will not lead to intrinsic motivation in the absence of autonomy [34]. Autonomy may be supported by helping the individual make decisions for personal reasons and helping them to make choices with minimal pressure [31-33]. Relatedness can be promoted by providing a sense of connectedness to others. An environment that helps a person feel socially included and supported by others may help facilitate intrinsic motivation [20,32,35].

Motivation for exercise is an important concept in the examination of cardiac rehabilitation adherence. Self-Determination Theory was previously used as a theoretical framework for motivational research in a cardiac rehabilitation setting [36]. Thorup and colleagues [36] showed qualitative evidence that a pedometer-based cardiac rehabilitation intervention supported autonomy, competence, and relatedness. It is possible that increasingly more self-determined (ie, internalized) motivation may be enough to help patients overcome the many obstacles associated with nonadherence to exercise and cardiac rehabilitation.

### Study Objectives

The purpose of the current randomized pilot trial is to determine the feasibility of using a Facebook intervention that provides education, peer support, and provider support to affect change in motivation and self-determination for exercise and adherence to cardiac rehabilitation in patients with CHD during a 12-week, Phase II cardiac rehabilitation program. It is hypothesized that the following will occur:

1. Scores for motivation for exercise overall will increase for patients exposed to a Facebook intervention and across individual motivational subtypes (ie, regulations) relative to a comparison group who receive educational handouts and emails.

2. The percentage of cardiac rehabilitation sessions attended will be higher relative to a comparison group who receive educational handouts and emails.

3. Engagement in the private Facebook group (ie, number of visits to the group and “likes”) will predict the number of cardiac rehabilitation sessions attended and the change in motivation. The feasibility of a larger trial will be based on sample size and participants’ engagement in the Facebook group.

## Methods

### Design

This is a prospective, randomized controlled pilot trial to evaluate the feasibility of using a social media intervention to affect change in motivation for exercise and adherence to cardiac rehabilitation sessions.

### Setting and Sample

The setting for this study will be in the outpatient cardiac rehabilitation at the main campus of a large tertiary care center in Northeast Ohio, USA, several satellite facilities in the region, and in patients’ homes or other locations where home computers might be accessed. This cardiac rehabilitation program provides electrocardiogram-monitored and supervised exercise, dietary guidance, and smoking cessation, behavioral counseling and stress reduction. All patients receive an individualized exercise prescription based on functional capacity at intake. Most patients, depending on insurance coverage, will be able to attend up to three sessions per week for a total of 36 sessions. In addition, patients are given guidance for unsupervised exercise at home.

All patients who are current and regular Facebook users, have qualified for cardiac rehabilitation (ie, diagnosed with CHD), and are entering cardiac rehabilitation at the main campus of this tertiary care center will qualify to participate in the study prior to beginning Phase II cardiac rehabilitation. Current Facebook users were chosen, as it is important that participants are skilled at using the Internet and familiar with social media. Regular use will be defined as logging on to Facebook at least two times in the last month. Inclusion criteria will include both men and women 18 years of age or older who speak English and live within 100 miles of the main campus of this tertiary care center. Participants must be able to read and understand English in order to complete the consent form, the Psychological Need Satisfaction in Exercise (PNSE) scale [37], and the Behavioral Regulation in Exercise Questionnaire-3 (BREQ-3). There will be no exclusion based on secondary diagnosis; however, participants must be able to exercise well enough to qualify to take part in cardiac rehabilitation.

### Measures

The primary hypothesis, change in motivation for exercise, will be measured at baseline and postintervention using the BREQ-3. The BREQ-3 is a 24-question validated instrument that measures forms of intrinsic and extrinsic regulation of exercise behavior [34] and is based on Self-Determination Theory. Psychometrics were first completed for the Behavioral Regulation in Exercise Questionnaire-2 (BREQ-2) by Markland and Tobin [38]. Cronbach alpha reliabilities were as follows: amotivation, .83; external regulation, .79; introjected regulation, .80; identified regulation, .73; and intrinsic regulation, .86. The BREQ-3 includes five questions in addition to those on the BREQ-2 and has a new subscale for integrated regulation [33]. The subscales (ie, regulations) of the BREQ-3 are used to calculate a relative autonomy index (RAI) [39]. Each question is answered on a 5-point Likert scale (range 0-4) and represents one of the regulations. The regulations are weighted then summed to give a single score. The resulting score or index gives an indication of the individual respondent’s self-determination for exercise. The RAI will place individual motivational subtypes or behavioral regulations on the self-determination continuum from amotivated (ie, lacking intention to exercise, score of -3) to intrinsically motivated (ie, self-determined or autonomously motivated, score of +3).

The PNSE scale will be used to assess need satisfaction with exercise. This scale was designed to assess the perception of psychological need satisfaction associated with self-determined motivation for exercise and consists of 18 items on a 6-point Likert scale, with three subscales measuring perceived competence, autonomy, and relatedness. The scale has shown high internal consistency (Cronbach alpha>.90) [37].

The secondary hypothesis, the percentage of cardiac rehabilitation sessions attended, will be measured at the time of cardiac rehabilitation completion or dropout. It will be calculated by dividing the number of sessions attended in a 3-month period of time by the total number of sessions allowed by insurance and multiplying by 100.

The tertiary hypothesis, Facebook engagement, will be assessed by measuring the number of “likes” by individuals on the private Facebook group. “Likes” (ie, the number of times a participant clicks “like” on any of the Facebook posts) will be counted and, along with visits to the group, will be used to examine the association between engagement in the social media intervention (ie, Facebook) and cardiac rehabilitation adherence and change in motivation. A postintervention questionnaire will be given to determine number of visits to the group. The participants will be asked to circle the number of times they accessed the private Facebook group per week: 0, 1-5, 6-10, 11-15, or >15 times. The questionnaire will also be used to collect qualitative data on participants’ perceptions of the intervention, including whether they felt supported in their care and more in touch with providers, whether or not they chatted with other Facebook members, and if the Facebook group affected their exercise behaviors. The questionnaire will use a 5-point Likert scale from 1 (*not at all*) to 5 (*quite a bit*) for all questions in addition to a section for comments. Participants may also grant permission for the evaluation of comments made on the private Facebook group, allowing the researchers to explore themes for qualitative analysis. Examination of comments will allow for a better understanding of the effectiveness of individual posts and the satisfaction of needs that may lead to self-determined motivation.

**Figure 2:**
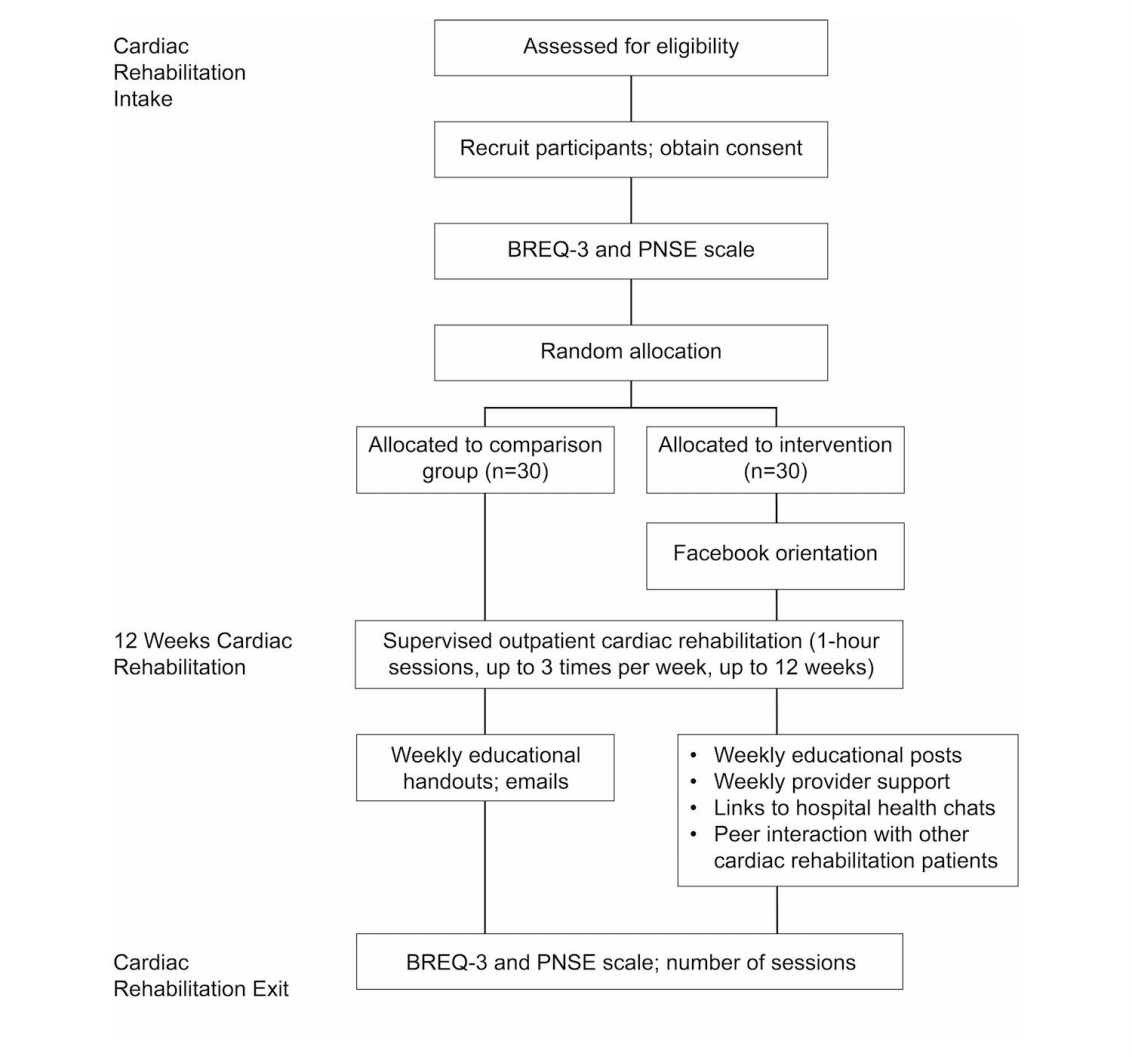
Study flowchart. BREQ-3: Behavioral Regulation in Exercise Questionnaire-3; PNSE: Psychological Need Satisfaction in Exercise.

Patient characteristics will be collected and will include key demographic variables (ie, age, gender, race, employment, distance to cardiac rehabilitation, and socioeconomic status), engagement (ie, number of visits to the group and “likes”), and key clinical variables (ie, cardiac rehabilitation indication, hypertension, diabetes, hyperlipidemia, and waist circumference), which will be obtained from the electronic medical record.

### Data Collection Procedures

#### Overview

Volunteers will be recruited from the main campus of this tertiary care center during their inpatient stay or the intake visit for cardiac rehabilitation at the main campus and satellite facilities in the region. Volunteers will be screened for Facebook use and interest in the study, the protocol will be explained, and volunteers will then be asked to sign consent forms. The consent form will address the fact that Facebook is a public forum and names and comments are seen by other participants and the research team. The Facebook group will be private in the sense that those not in the group will not be able to see the content. Participants will complete a baseline BREQ-3 questionnaire and PNSE scale at the time of consent. Participants will then be randomized to Facebook versus comparison groups using blocked randomization (see Figure 2).

#### Intervention

The Facebook intervention will include peer support, education, provider support, and text message prompts when new posts are added. These interventions are designed to minimize pressure, offer choices, and allow for peer interaction, positive feedback, guidance, and direction in order to provide support for competence, autonomy, and relatedness. Competence will primarily be supported with the use of educational posts in the Facebook group. Autonomy support will come from the provider posts. Finally, relatedness will be supported by peer interaction and engagement in the Facebook group.

Educational posts will cover 12 topics that will encourage participants to practice preventive heart care while offering a variety of suggestions and encouragement for making personal health care choices. The educational portion of the intervention is designed to offer clear information and structure, thus supporting competence, which may help to enhance intrinsic motivation. These 12 educational topics will be standardized such that they will be posted on the Facebook group, one each week, and then the same ones will be reposted again every 12 weeks. The posts may be in the form of text, video, and/or pictures; they will include materials from the hospital’s health library and other fact sheets and videos produced by the hospital, the American Heart Association, and the US Centers for Disease Control.

Provider posts will include topics such as motivational quotes, encouragement, reminders to exercise independently, and reminders to contact providers with questions. These postings are designed to promote a sense of choice and help participants feel that providers see them as having a unique frame of reference, thus being supportive of autonomy. Providers will be nurses on the research team, exercise physiologists, nurse practitioners, and physicians who may or may not choose to reveal their personal identities. All Facebook participants will see the same content. Provider support will also include links to provider health chats, in which patients can chat online with providers at set dates and times.

Peer interaction on Facebook will be as frequent as the participant freely chooses and will be monitored daily by the research team for appropriateness of content. Engagement in Facebook is designed to offer an opportunity for social inclusion and a sense of involvement, allowing for relatedness.

The comparison group will receive the same educational and provider support materials as the Facebook group, but will receive it in the form of a handout or via email in the event the patient cannot be contacted or misses cardiac rehabilitation on a particular week. Both groups will have the opportunity for weekly education classes and typical peer interactions, which will involve up to 3 hours of group cardiac rehabilitation per week.

Upon cardiac rehabilitation completion or dropout, post data will be collected. It is anticipated that this pilot will take up to 1 year and will be completed when 30 participants for each group have been obtained (see Table 1).

**Table 1:** Study calendar.

	Study timeline				
	Month 1	Months 2-7	Months 4-11	Months 4-12	Month 12
Study event	Begin recruitment; Complete intake for first 8 subjects (BREQ-3^a^, PNSE^b^ scale); No data used for first 8 subjects	Collection of intake data	Collection of exit data	Data cleaning	Statistical analysis; Begin manuscript writing and preparation for longer trial

^a^BREQ-3: Behavioral Regulation in Exercise Questionnaire-3.

^b^PNSE: Psychological Need Satisfaction in Exercise.

### Data Analysis

#### Statistical Methods

This is a feasibility study and the sample size obtained will determine if the study is appropriately powered to detect the desired effect size. Patient characteristics will be summarized by group using frequencies and percentages for categorical factors, and using means and standard deviations for continuous measures. In order to examine the primary outcome—differences in change in motivation between groups—overall motivation using the RAI from the BREQ-3 will be evaluated using analysis of covariance (ANCOVA) models. Mean differences with 95% CIs for group differences will be presented. Multivariate analysis of variance models will be used to evaluate differences in the change across individual motivation subtypes (ie, regulations), using the BREQ-3, between groups overall. If significant, separate ANCOVA models for each subtype will be fit. Similar ANCOVA models will be used to compare changes in needs satisfaction scores, overall and separately among the three subscales, between groups. Two-sample *t* tests will be used to compare number of sessions completed. As a secondary analysis, the relationships between patient characteristics, numbers of visits to the group and “likes,” and the outcome variables—RAI change, number of sessions, and needs satisfaction change—will be examined using *t* tests and Pearson correlations. The correlation between changes in RAI and needs satisfaction will also be evaluated. Analyses will be performed using SAS version 9.4 software (SAS Institute Inc). An overall significance level of .05 will be assumed for all tests.

#### Sample Size

The investigators plan to enroll 30 patients in each group. In the first 9 months of 2016, cardiac rehabilitation at the main campus of this tertiary care center had approximately 170 patient intakes. It is assumed that there will be a similar number of patient intakes for a 9-month period in 2017. Based on Facebook participation rates for those over 50 years of age [21] and the high participation rates in previous research projects in this facility’s cardiac rehabilitation center, it is estimated that 40% may meet eligibility requirements and agree to participate.  Allowing for use of the first 8 participants to establish the Facebook group, the estimated sample size would then be 60 total participants for randomization to study groups who can then be included in analysis.  With this sample size, there will be 86% power to detect large effect sizes (Cohen *d*=0.8) for our study outcomes [40]. The primary aims of this sample size determination is to evaluate whether the proposed intervention is feasible and to estimate the differences that might exist so that a larger trial that would have adequate power to detect smaller differences could be designed based on what was learned in this pilot study. The sample size of 30 per group was chosen primarily to facilitate a large intervention group, since the value of the intervention is predicated upon interaction among the participants.

### Human Subjects Protection

This feasibility study has been approved by the Institutional Review Board of this tertiary care center (Study No. 16-1456) and is registered at ClinicalTrials.gov (NCT02971813).

Participants will be assured that participation in the study at all times is voluntary and will not affect their care in any way. Protection of human subjects for this study will be further ensured through the process of informed consent. Participants in the intervention and comparison groups will be informed that privacy of medical information will be ensured. However, due to the nature of social media, information or comments posted by the patients in the Facebook group will be visible to others in the group as well as the study team. For this reason, the informed consent will address the fact that comments may be seen by others.

All responses from participants on the Facebook group will be assigned a number and all other identifying information will be removed for data analysis. Any data on paper will be kept in the principal investigator’s (LAS) locked office in a locked filing cabinet. All electronic data will be stored on the principal investigator’s computer, which requires password entry, in a folder accessible only to the principal investigator and the research team, and on an encrypted thumb drive. Dissemination of findings will be deidentified and reported numerically in narrative form or in aggregate, with no personal identifiers.

## Results

This project was funded in July 2017 and recruitment is currently underway. The recruitment goal is 60 cardiac rehabilitation patients. Data collection is anticipated to be complete by July 2018.

## Discussion

### Overview

The main objective of this project will be to examine the feasibility of a novel Facebook intervention to address patient adherence to cardiac rehabilitation. Improving uptake and adherence to cardiac rehabilitation is of paramount importance in the secondary prevention of CHD. This study will use the validated BREQ-3 and the PNSE scale and examine the effect of a Facebook intervention on number of cardiac rehabilitation sessions attended. Applying the Self-Determination Theory, the research team will provide educational and provider support postings on a private Facebook group. The participants will have the opportunity to learn and interact with other participants in this social media platform. This study has the potential to affect a change in patient motivation for exercise and cardiac rehabilitation adherence, thus reducing complications and hospital readmissions among patients eligible for cardiac rehabilitation.

### Limitations and Unanticipated Problems

Limitations for this study include the variable number of sessions paid for by non-Medicare and non-Medicaid insurance. This could potentially affect motivation or participation in the Facebook group and cardiac rehabilitation itself if the patient has few sessions that are covered by insurance. Feasibility concerns for the pilot include obtaining a large enough cohort of patients in order to have peer support, especially for those who enroll in the early stages of the study. Data will not be included for the first 8 participants in order to ensure that there is a large enough group of Facebook users to enable social networking among participants. Additionally, patient visits to the Facebook group rely on self-report and are therefore subject to reporting bias.

There are limitations to this feasibility study that can be addressed in a larger trial. Patients who are not current Facebook users were excluded from the pilot trial. If Facebook is demonstrated to be a feasible venue for presenting and testing motivation for exercise, those who are not currently on Facebook should be included in a larger trial. The fact that patients may see each other in cardiac rehabilitation sessions presents a potential for diffusion bias, demoralization, or rivalry. This has been minimized to the extent that few participants are likely to communicate about the study in cardiac rehabilitation sessions due to the number of classes and facilities; however, it will need to be a consideration for this and larger studies.

### Conclusions

The findings of this study will help to determine the feasibility of using a Facebook intervention to affect adherence and motivation. It has the possibility of opening doors to other technological interventions and unique approaches to improving health outcomes in this population. The results of this study will determine if a larger-scale intervention is feasible. Further, this pilot study will be the first to examine the effect of a Facebook intervention on patient adherence and motivation for exercise in a cardiac rehabilitation setting. The established private, cardiac rehabilitation Facebook group will enable a larger-scale intervention to be implemented and will allow for the examination of additional outcome variables. This intervention has the potential to add innovative approaches to the body of evidence seeking ways to improve patient outcomes in cardiac rehabilitation.

## Abbreviations

ANCOVA: analysis of covariance
BREQ-2: Behavioral Regulation in Exercise Questionnaire-2
BREQ-3: Behavioral Regulation in Exercise Questionnaire-3
CHD: coronary heart disease
PNSE: Psychological Need Satisfaction in Exercise
RAI: relative autonomy index
